# Fortified Pasta With Cricket (*Acheta domesticus*) Powder: Impact of an Alternative Protein Source on Pasta Nutritional, Functional, and Technological Properties

**DOI:** 10.1111/1750-3841.70861

**Published:** 2026-01-18

**Authors:** Leonardo Musto, Mussa Makran, Dario Mercatante, Ivan Albano, Maria Teresa Rodriguez‐Estrada, Antonio Cilla, Guadalupe Garcia‐Llatas

**Affiliations:** ^1^ Nutrition and Food Science Area, Faculty of Pharmacy and Food Sciences University of Valencia Valencia Spain; ^2^ Department of Agricultural and Food Sciences Alma Mater Studiorum‐Università di Bologna Bologna Italy; ^3^ Italian Cricket Farm Scalenghe Italy; ^4^ CIRI‐Agrifood (Interdepartmental Centre of Industrial Agrifood Research) Alma Mater Studiorum‐Università di Bologna Cesena Italy

**Keywords:** entomophagy, glycemic index, INFOGEST 2.0, physicochemical evaluation, total antioxidant capacity, total fatty acid profile

## Abstract

Global population growth is challenging the sustainability of current food systems and driving the search for alternative protein sources with lower environmental impact. The house cricket (*Acheta domesticus*) has emerged as a promising, sustainable provider of high‐quality protein. This study assessed, for the first time, starch hydrolysis kinetics and antioxidant capacity of *penne rigate* pasta formulated with 10% cricket powder versus 100% durum wheat pasta after a simulated gastrointestinal digestion model (INFOGEST 2.0). Glucose release was monitored throughout digestion, whereas antioxidant capacity was evaluated in raw and cooked pasta, as well as in bioaccessible and non‐bioaccessible fractions, using oxygen radical absorbance capacity (ORAC), Trolox equivalent antioxidant capacity (TEAC), and Folin–Ciocalteu assays. Nutritional composition and technological properties were also analyzed. Cricket‐enriched pasta followed first‐order starch hydrolysis kinetics, in contrast to the linear kinetics observed in 100% durum wheat pasta. It showed significantly lower starch hydrolysis (61.5% vs. 85.3%) and higher resistant starch content (38.5% vs. 14.7%). Antioxidant capacity generally increased due to cricket‐enrichment and gastrointestinal digestion. The enriched pasta also exhibited increased protein (26.5%), lipid (98.4%, showing higher polyunsaturated and saturated fatty acids proportion), dietary fiber (89.0%), and mineral contents (15.9%) but decreased carbohydrate (13.5%) and starch levels (11.1%). No significant differences were found in water absorption or cooking loss. Color was notably darker, being significantly different from 100% durum wheat pasta (Δ*E*_Lab_ of 19.34). Overall, in vitro digestion results suggest that cricket‐enriched pasta may contribute to improved glycemic response modulation and antioxidant intake in sustainable dietary strategies, without altering the technological properties of the pasta.

## 1 Introduction

Increasing the consumption of insect‐based foods is considered a good strategy for mitigating the negative effects of climate change (Imathiu 2020). Life cycle assessment studies demonstrate that insect protein production requires significantly less land and generates substantially lower greenhouse gas emissions compared to conventional animal proteins such as chicken, pork, and beef (Oonincx and de Boer 2012; Vinci et al. 2022). In particular, research indicates that cricket (*Acheta domesticus*) farming offers considerable environmental advantages over broiler production, requiring 50% less water and 20% less feed per kg of edible protein (Halloran et al. 2017). Beyond environmental benefits, entomophagy also offers nutritionally valuable alternatives to conventional meat. Many edible insect species provide proteins of comparable biological quality, being particularly rich in branched‐chain amino acids, such as leucine, isoleucine, and valine (Udomsil et al. 2019; Orkusz 2021). Moreover, insect proteins exhibit high digestibility, with absorption rates ranging from 76% to 96% (Kouřimská and Adámková 2016). Thus, their complete essential amino acid profile combined with high digestibility positions edible insects as a protein source of high biological value for human consumption. In addition to their protein quality, edible insects exhibit more favorable polyunsaturated to saturated fatty acid (FA) (PUFA/SFA) ratios than most traditional meat products. Furthermore, edible insects are a notable source of dietary fiber, a nutrient typically lacking in conventional animal‐derived foods (Orkusz 2021). As a result of these environmental and nutritional advantages, several insect species, including *A. domesticus*, have been approved as novel foods within the European Union (Siddiqui et al. 2024).

Despite these advantages, consumer acceptance of insect‐based foods remains a significant barrier in Western countries. A recent Italian survey (*n* = 200) revealed very low social acceptance, even among individuals who were more informed about sustainable diets (Simeone and Scarpato 2021). Nevertheless, acceptance tends to increase when insects are incorporated in non‐visible forms, as food ingredients (Ranga et al. 2025). Given that wheat pasta is a widely consumed staple food in many traditional diets (Webb 2019), fortifying pasta with insect powder represents a promising strategy to introduce insect‐based products into non‐entomophagous societies. In this regard, several studies have already reported favorable consumer responses to cricket‐enriched pasta (Duda et al. 2019).

Although consumer interest is growing, the functional properties of crickets and cricket‐enriched pasta have been scarcely explored. Some preclinical studies on another cricket species (*Gryllus bimaculatus*) have demonstrated antioxidant (Hwang et al. 2019) and anti‐inflammatory properties (Kim et al. 2021). In the case of *A. domesticus*, total antioxidant capacity has been assessed in a few studies (Lucas‐González et al. 2019; Di Mattia et al. 2019). Regarding *A. domesticus*‐enriched pasta, only one study to date has examined its antioxidant potential, reporting that the enrichment can enhance this property (Jakab et al. 2020). However, this study only evaluated the in vitro antioxidant capacity of pasta, without simulated digestion. Gastrointestinal digestion can modify the bioactivity of such compounds; thus, simulating gastrointestinal digestion is critical to better understand the actual in vivo potential of cricket‐enriched products (Dima et al. 2020). As oxidative stress is implicated in chronic diseases such as diabetes *mellitus* and cardiovascular disorders, and dietary antioxidants contribute to their prevention (Zielińska et al. 2021), enhancing antioxidant intake through cricket‐based foods may offer health benefits.

Despite the potential benefits of cricket enrichment, refined grain products like pasta are known to rapidly increase postprandial blood glucose due to fast starch digestion and absorption. A large prospective cohort study involving 148,858 participants across 21 countries found that high refined grain consumption was associated with a 27% increase in total mortality and a 33% higher risk of major cardiovascular events. Glycemic response was identified as one of the mechanisms behind these associations (Swaminathan et al. 2021). Conversely, whole grain intake is associated with a 19% reduction in all‐cause mortality (Reynolds et al. 2019). Therefore, it is of particular interest to assess whether cricket enrichment can positively modulate the glycemic response of refined pasta. In this context, evaluating starch hydrolysis during in vitro simulated digestion provides a reliable method to estimate the glycemic impact of foods.

To our knowledge, no study has yet investigated the effect of *A. domesticus*‐enriched pasta on in vitro starch hydrolysis kinetics or antioxidant behavior following simulated gastrointestinal digestion, highlighting a notable gap in the current literature. Therefore, the aim of this study was to evaluate the effects of *A. domesticus* powder enrichment in durum wheat pasta on in vitro starch hydrolysis kinetics and antioxidant capacity after simulated gastrointestinal digestion. Additionally, the nutritional composition and several technological properties of the pasta were assessed. This study represents the first investigation into the starch hydrolysis kinetics and antioxidant properties of cricket‐enriched pasta under simulated digestion conditions, providing novel insights into its potential health benefits.

## 2 Methodology

### 2.1 Reagents

Analytical‐grade solvents (e.g., methanol, chloroform, ethyl acetate, diethyl ether, acetone, and *n*‐hexane), derivatization reagents (sodium methoxide and boron trifluoride), FA methyl ester (FAME) standard (methyl tridecanoate), and other common laboratory chemicals (sodium chloride, potassium chloride, disodium tetraborate decahydrate, sodium sulfate, and glycerol) were obtained from Sigma‐Aldrich (St. Louis, MO, USA). Reference standard mixture of FAME GLC‐463 was purchased from Nu‐Chek Prep Inc. (MN, USA). 2,2′‐Azinobis(3‐ethylbenzothiazoline‐6‐sulfonic acid) (ABTS), 2,2′‐azobis(2‐methylpropionamidine) dihydrochloride (AAPH), Folin–Ciocalteu reagent, gallic acid, 6‐hydroxy‐2,5,7,8‐tetramethylchroman‐2‐carboxylic acid (Trolox), potassium persulfate, sodium carbonate, sodium fluorescein, α‐amylase from human saliva (E.C. 3.2.1.1) (70.4 U/mg), bovine bile (1.24 µmol/mg), calcium chloride dihydrate, pancreatin from porcine pancreas (10.3 U/mg), and pepsin from porcine gastric mucosa (E.C. 3.4.23.1) (3240 U/mg) were obtained from Merck LifeScience S.L.U. (Madrid, Spain). Rabbit gastric extract (RGE) (lipase: 16 U/mg; pepsine: 394 U/mg) was acquired from Lipolytech (Marseille, France). Glucose quantification reagents (GOPOD) kit (K‐GLUC 09/14), as well as amyloglucosidase solution, were provided by Megazyme Ltd. (Bray, Co., Wicklow, Ireland). Ultrapure water (18.2 MΩ cm) was obtained using a Milli‐Q water purification system (Millipore, Bedford, MA, USA).

### 2.2 Samples

Experimental samples consisted of *penne rigate*, a traditional Italian short pasta shape. The insect‐enriched pasta was supplied by Italian Cricket Farm (Scalenghe, Turin, Italy). The cricket‐enriched pasta was prepared using durum wheat flour, 10% of cricket (*A. domesticus*) powder and water. The dough was mixed in an orbital mixer until a homogeneous consistency was achieved, then extruded through a bronze die to obtain the desired *penne rigate* shape. Drying was carried out at 40°C for 24 h.

Additionally, a commercial reference from a different supplier was included for comparison: A conventional 100% durum wheat pasta obtained from a local supermarket (Valencia, Spain).

### 2.3 Proximate Composition and Starch Content

The proximate composition of 100% durum wheat pasta and cricket powder‐enriched pasta was analyzed. Prior to analysis, both pasta samples were ground into a fine powder. Moisture and ash contents were determined according to American Association of Cereal Chemists (AACC) (2010) Methods 44‐19.01 and 08‐12.01, respectively. Total nitrogen was measured using the Kjeldahl method (AACC Method 46‐12.01, 2010), and protein content was calculated by multiplying the nitrogen value by a conversion factor of 6.25. Fat content was quantified via Soxhlet extraction following Association of Official Analytical Collaboration (AOAC) Official Method 14.059 (1990). Total dietary fiber was measured using an enzymatic‐gravimetric method with a commercial assay kit, following AACC Method 32‐05.01 (2010). Total starch content was assessed in accordance with AOAC Official Method 996.11 (1996). Carbohydrate content was estimated by difference, subtracting the measured values of moisture, fat, protein, ash, and dietary fiber from 100%. All results were expressed as g/100 g of raw sample on a wet basis. Detailed procedures for proximate analysis determination can be found in the Supporting Information.

### 2.4 Pasta Cooking

Cooking conditions were established following the AACC Method 66‐50 (American Association of Cereal Chemists International 2000), which assesses pasta and noodle cooking quality—firmness. A volume of 300 mL of distilled water was added to a 500‐mL beaker and brought to a rolling boil on a hot plate. Once boiling, 25 g of each pasta sample were introduced into the water, ensuring a water‐to‐sample ratio of at least 10:1 throughout the cooking process. Preliminary cooking trials were performed to determine the optimal cooking time. According to the specified method, beginning at 10 min from the start of cooking, a pasta strand was removed every 30 s and compressed between two transparent plastic sheets. The optimal cooking time was defined as the point at which the opaque core of the pasta was no longer visible. On the basis of these trials, a final cooking time of 10 min was established for sample enriched with cricket powder and 11 min for sample 100% durum wheat.

### 2.5 Lipid Extraction

Lipid extraction from pasta samples was performed using a modified Folch method, as described by Boselli et al. (2001). Approximately 25 g of each sample were extracted using a chloroform:methanol mixture (1:1, v/v), followed by the addition of other 100 mL of chloroform. Subsequently, 1 M potassium chloride was added to facilitate phase separation. The organic phase was collected and evaporated to dryness with a rotavapor. The total lipid content was then determined gravimetrically and data were expressed as g of lipids/100 g of raw sample on a wet basis. All extractions were performed in triplicate for each sample.

### 2.6 Determination of FA Composition

FA composition of pasta samples was determined through a double methylation procedure in a methanolic medium, involving a sequential treatment with sodium methoxide followed by boron trifluoride, to ensure complete methylation of all FAs, including free fatty ones (Varona et al. 2021). Approximately 20 mg of lipid extract were weighed and spiked with tridecanoic acid methyl ester (C13:0) as an internal standard. After methylation, 1 µL of the resulting solution containing FAME was injected into a gas chromatograph equipped with a flame ionization detector (GC‐FID, Shimadzu, Kyoto, Japan), using the analytical conditions reported by Mandrioli et al. (2024). Individual FAs were identified by comparing their retention times with those of a commercial FAME standard mixture. GC response factors were calculated for each FA using the standard mix and internal standard (C13:0). Quantification was performed using the internal standard method, and results were expressed as g/100 g of total FAME. All analyses were conducted in triplicate.

### 2.7 In Vitro Gastrointestinal Digestion

Starch digestibility was evaluated through simulated gastrointestinal digestion following the standardized INFOGEST 2.0 protocol (Brodkorb et al. 2019). Prior to digestion, cooked durum wheat and cricket‐enriched pasta samples were pre‐ground using a mechanical grinder to achieve a particle size comparable to that generated by mastication, thereby addressing limitations in replicating the in vivo oral phase mechanically (Miedes et al. 2023). During the oral phase, 5 g of ground pasta or ultrapure water (used as a digestion blank) were mixed with 3.5 mL of simulated salivary fluid and manually shaken for 1 min. Subsequently, 0.5 mL of α‐amylase solution (1500 U/mL; final concentration: 75 U/mL in the oral digesta), 25 µL of 0.3 M CaCl_2_, and 975 µL of ultrapure water were added to reach a final volume of 10 mL. The mixture was incubated at 37°C in a shaking water bath (95 rpm) for 2 min. For the gastric phase, 7.5 mL of simulated gastric fluid, 1.6 mL of pepsin solution (25,000 U/mL; final concentration: 2000 U/mL), 0.98 mL of RGE solution (225 U/mL; final concentration: 60 U/mL), and 5 µL of 0.3 M CaCl_2_ were added. The mixture was manually stirred for 1 min, the pH was adjusted to 3, and ultrapure water was added to reach a final volume of 20 mL. The samples were then incubated at 37°C in a shaking water bath (95 rpm) for 2 h. In the intestinal phase, 11 mL of simulated intestinal fluid, 5 mL of pancreatin solution (800 U/mL based on trypsin activity; final concentration: 100 U/mL), 40 µL of 0.3 M CaCl_2_, and 2.5 mL of bile extract solution (containing 166 mM bile salts; final concentration: 10 mM) were added. The mixture was stirred manually for 1 min, the pH was adjusted to 7, and ultrapure water was added to reach a final volume of 40 mL. The samples were incubated at 37°C and 95 rpm for other 2 h. After digestion, the mixtures were centrifuged at 3100 × *g* for 90 min at 4°C. The supernatant, representing the bioaccessible fraction (BF), was separated, whereas the remaining pellet was defined as the non‐BF (NBF). Each sample was subjected to in vitro digestion in triplicate (three biological replicates). Both the samples and the digestion blanks were digested under the same conditions.

### 2.8 Starch Hydrolysis Assessment

Starch hydrolysis was assessed according to the method described by Makran et al. (2023). Glucose release was quantified in undigested pasta samples as well as in oral, gastric, and intestinal digesta using the GOPOD assay. Samples from the intestinal phase were collected at multiple time points (0, 10, 20, 40, 60, 80, 100, and 120 min). Sample preparation varied depending on the digestion phase. For undigested pasta and oral digesta, a preliminary aqueous extraction was conducted: 1 or 2 g of ground pasta or oral digesta was mixed with 6 or 7 mL of ultrapure water, respectively. The mixtures were centrifuged at 3600 × *g* for 30 min, and 100 µL of the resulting supernatant was combined with 400 µL of ethanol. In contrast, gastric and intestinal digesta were processed without prior water extraction; 100 µL of digesta was directly mixed with 400 µL of ethanol. All samples were then incubated under agitation for 30 min using a sample tube rocker and centrifuged at 4000 × *g* for 15 min. Subsequently, 100 µL of the supernatant from each sample was incubated with 500 µL of amyloglucosidase solution (27 U/mL in sodium acetate buffer, pH 4.8) at 37°C for 1 h. Then, 10 µL of the resulting reaction mixture was transferred to a 96‐well microplate, followed by the addition of 290 µL of GOPOD reagent. The plate was incubated at 50°C for 30 min, and absorbance was measured at 510 nm. The starch digestion rate was expressed as the percentage of total starch hydrolyzed at each time point. Kinetics of intestinal starch hydrolysis were modeled using either a linear zero‐order equation (*C_t_ = C*_0_ *+ kt*) or a nonlinear first‐order equation (*C_t_ = C*_0_ *+ C*_∞_ [*1 − *e^−^ *^kt^*]) where *C_t_* is the percentage of starch hydrolyzed at time t, *C*_0_ is the percentage hydrolyzed during the orogastric phase, and *C*_∞_ is the total hydrolyzed by the end of the intestinal phase. For the linear model, *k* represents the hydrolysis rate (percentage of starch hydrolyzed per min), whereas for the first‐order model, *k* is the rate constant (min^−^^1^). Resistant starch was calculated as the percentage of starch remaining unhydrolyzed after completion of the intestinal phase.

### 2.9 Antioxidant Capacity

The antioxidant capacity of pasta samples was assessed before and after cooking, as well as after in vitro gastrointestinal digestion. Three complementary assays were employed to evaluate distinct antioxidant mechanisms: the oxygen radical absorbance capacity (ORAC) method to assess hydrogen atom transfer, the Folin–Ciocalteu assay to determine electron transfer capacity, and the Trolox equivalent antioxidant capacity (TEAC) test to evaluate a mixed mode of antioxidant action (Prior et al. 2005). An analytical triplicate was conducted for non‐digested samples, whereas a biological triplicate was performed for digested samples.

#### 2.9.1 Extraction of Antioxidant Compounds

Briefly, 2 g of ground raw pasta or 5 g of cooked homogenized pasta were mixed with 10 mL of 80% (v/v) methanol. The mixtures were incubated at approximately 28°C on an orbital shaker at 180 rpm for 2 h. Following incubation, the samples were centrifuged at 1400 × *g* for 20 min, and the supernatants were collected and stored. The remaining residues were subjected to a second extraction under identical conditions. Supernatants from both extraction steps were pooled for subsequent antioxidant capacity analysis (Gull et al. 2018).

To evaluate the impact of in vitro gastrointestinal digestion on antioxidant capacity, samples were analyzed in both the BF and the NBF. The BF was analyzed without prior extraction, whereas the NBF underwent the same extraction procedure described above, using the entire residue resulting from the digestion process. To account for any potential antioxidant contribution from reagents used during simulated gastrointestinal digestion, a digestion blank was analyzed and its values subtracted from sample results.

#### 2.9.2 ORAC Assay

The ORAC test followed established methodologies (Cilla et al. 2011). In brief, each well of a white microplate received 80 µL of sodium fluorescein (0.015 mg/mL), 80 µL of the test sample (diluted 1:250 v/v in 75 mM phosphate buffer at pH 7.4), and 40 µL of AAPH solution (120 mg/mL). Fluorescence was monitored every 5 min for a total of 90 min at excitation/emission wavelengths of 485/520 nm using a Victor^3^ Multi‐Label Counter (Perkin‐Elmer, Waltham, MA, USA). The antioxidant capacity was quantified by calculating the area under the fluorescence decay curve and expressed as mmol of Trolox equivalents per 100 g of raw pasta on a wet basis, based on a Trolox calibration (20 µM).

#### 2.9.3 TEAC Assay

The TEAC assay was carried out as described by Cilla et al. (2011). A stock of ABTS solution was prepared by mixing 6.9 mM ABTS with 2.4 mM potassium persulfate and incubating (16 h) the mixture at room temperature in the dark. This solution was then diluted with ethanol to reach an absorbance of 0.70 ± 0.02 at 734 nm (measured at 30°C) (Lambda 365 UV/vis spectrophotometer, Perkin‐Elmer, Waltham, MA, USA). For the assay, 2 mL of the diluted ABTS solution was mixed with 100 µL of either the sample (undigested samples and NBF without dilution and BF diluted 1:5 v/v) or a Trolox standard (100–1000 µM). Absorbance was recorded after a 3‐min incubation, and the percentage of radical inhibition was calculated. Results were reported as mmol Trolox equivalents per 100 g of raw pasta on a wet basis.

#### 2.9.4 Folin–Ciocalteu Reducing Capacity

This method was adapted from Cilla et al. (2011). A volume of 10 µL of each sample was combined with 300 µL of 2% (w/v) sodium carbonate and 10 µL of 1 N Folin–Ciocalteu reagent in a 96‐well plate. The mixture was incubated for 1 h at room temperature in the absence of light. Absorbance was then read at 765 nm using a microplate reader (FLUOstar Omega, Bmg Labtech, Germany). Antioxidant capacity was calculated as gallic acid equivalents (GAE) per 100 g of raw pasta on a wet basis, using a standard curve prepared with gallic acid concentrations ranging from 10 to 300 mg/L.

### 2.10 Technological Properties

#### 2.10.1 Evaluation of Culinary Properties

Culinary quality parameters, specifically water absorption capacity (WA) and cooking loss, were determined according to AACC Method 66‐50.01.

#### 2.10.2 Colorimetric Analysis

Pasta color measurements were conducted using a ColorQuest XE colorimeter (HunterLab, Leicestershire, United Kingdom), following the methodology outlined by Ho et al. (2022). In this system, the L* value denotes lightness (ranging from 0 [black] to 100 [white]), the a* value represents the red–green axis (with negative values indicating green and positive values denoting red), and the b* value corresponds to the blue–yellow axis (negative values indicate blue and positive values denote yellow). Additionally, the color differences between samples were estimated using the Δ*E*_Lab_ calculation.

#### 2.10.3 Image Analysis

The appearance of the samples was assessed using an “electronic eye” system (visual analyzer VA400 IRIS, Alpha MOS, France), equipped with a high‐resolution CCD (charge‐coupled device) camera (2592 × 1944 pixels) and advanced data processing software. The procedure followed the method described by Barbieri et al. (2021). This system enables discrimination among samples based on attributes such as shape, size, color intensity, and color uniformity. The image analysis was conducted on pasta samples made with cricket powder and durum wheat, before and after cooking.

### 2.11 Statistical Analyses

Data were expressed as mean ± standard deviation from at least three independent replicates. The data were analyzed using Statgraphics Plus 5.1 software (Statpoint Technologies Inc., Warrenton, VA, USA). Statistical analysis was performed using independent *t*‐tests applied separately to each measured parameter to compare the means of selected variables between the cricket‐enriched pasta and the 100% durum wheat pasta. To evaluate starch hydrolysis, an ANOVA followed by Tukey post hoc test was performed to determine significant differences across the time points of the in vitro digestion. A significance level of *p* < 0.05 was considered statistically significant.

## 3 Results and Discussion

### 3.1 Nutritional Composition

**FIGURE 1:**
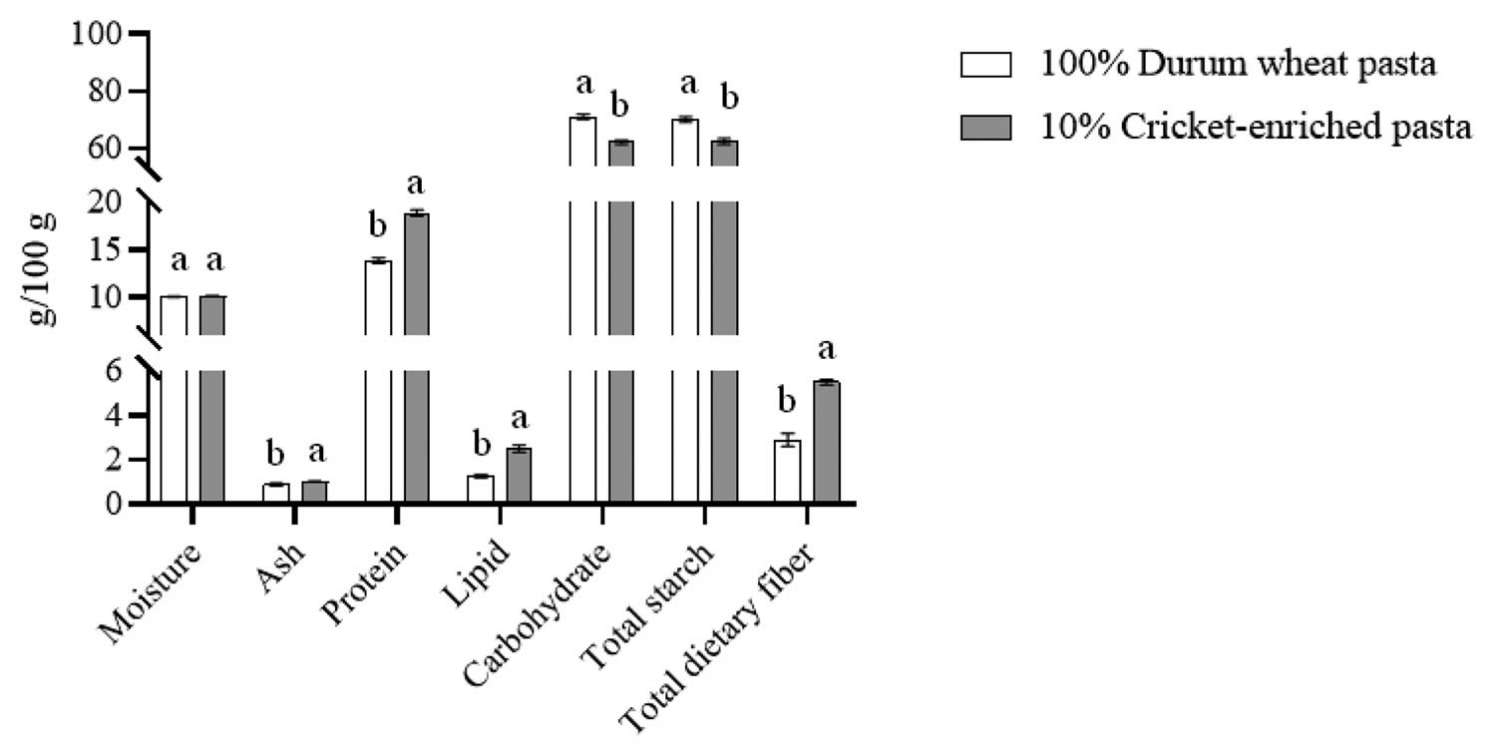
Proximate composition (g/100 g raw pasta) of durum wheat and cricket‐enriched pasta. Different letters indicate significant differences (*p* < 0.05) between pasta types. Data are expressed as mean ± standard deviation (*n* = 3 analytical replicates).

The proximate composition of durum wheat pasta and cricket‐enriched pasta is shown in Figure 1 (as well as in Table S1). The addition of cricket powder did not significantly affect the moisture content *(p* > 0.05) compared to durum wheat pasta (10.10 vs. 10.08 g/100 g). As shelf‐stable pasta undergoes a drying process during manufacturing, it is essential to control how added ingredients, such as cricket powder, influence drying, as this can directly affect the final moisture content and thus the shelf‐life (Ribeiro et al. 2021). Although Jakab et al. (2020) report changes in moisture content in pasta formulated with cricket powder, others observe no significant differences (Ho et al. 2022). These discrepancies may be attributed to differences in cricket species, enrichment percentage, and cereal type (Duda et al. 2019; Jakab et al. 2020). For instance, Jakab et al. (2020) used millet flour enriched with 5%–10% *G. bimaculatus* powder, whereas Ho et al. (2022), like the present study, utilized durum wheat flour enriched with 5% *A. domesticus* powder.

On the other hand, the ash content was significantly higher (*p* < 0.05) in the cricket‐enriched pasta than in the control (1.02 vs. 0.88 g/100 g), being consistent with previous findings (Duda et al. 2019). Cricket powder (*A. domesticus*) is notably rich in minerals (mg/100 g dry weight), particularly phosphorus (899), potassium (389), calcium (149), magnesium (136), and zinc (19) (Udomsil et al. 2019). Thus, incorporating cricket powder into refined cereal‐based foods, which are generally mineral‐deficient (Zingale et al. 2023), may significantly enhance their micronutrient profile and dietary value.

Similarly, the protein content increased significantly (*p* < 0.05) in the cricket‐enriched pasta compared to the control (18.87 vs. 13.88 g/100 g), a result aligned with findings from other studies (Duda et al. 2019; Ho et al. 2022). Insects generally provide high‐quality protein. Orkusz (2021) analyzed the amino acid composition of various insects and found that they deliver proteins with an aminoacidic profile comparable to traditional meat sources. Cricket powder (*A. domesticus*), in particular, is rich in essential amino acids (42.7% of total amino acids), with high contents (g/100 g) of leucine (3.8), isoleucine (2.9), and valine (4.5) (Udomsil et al. 2019). It also contains lysine (3.2) and threonine (1.7), which are deficient in wheat proteins (Filip and Vidrih 2015), making cricket powder an effective enrichment ingredient. In terms of protein digestibility, insect proteins demonstrate absorption rates of 76%–96% (Kouřimská and Adámková 2016), but they can be lower if bound to chitin in the insect exoskeleton (Rumpold and Schlüter 2013).

**FIGURE 2:**
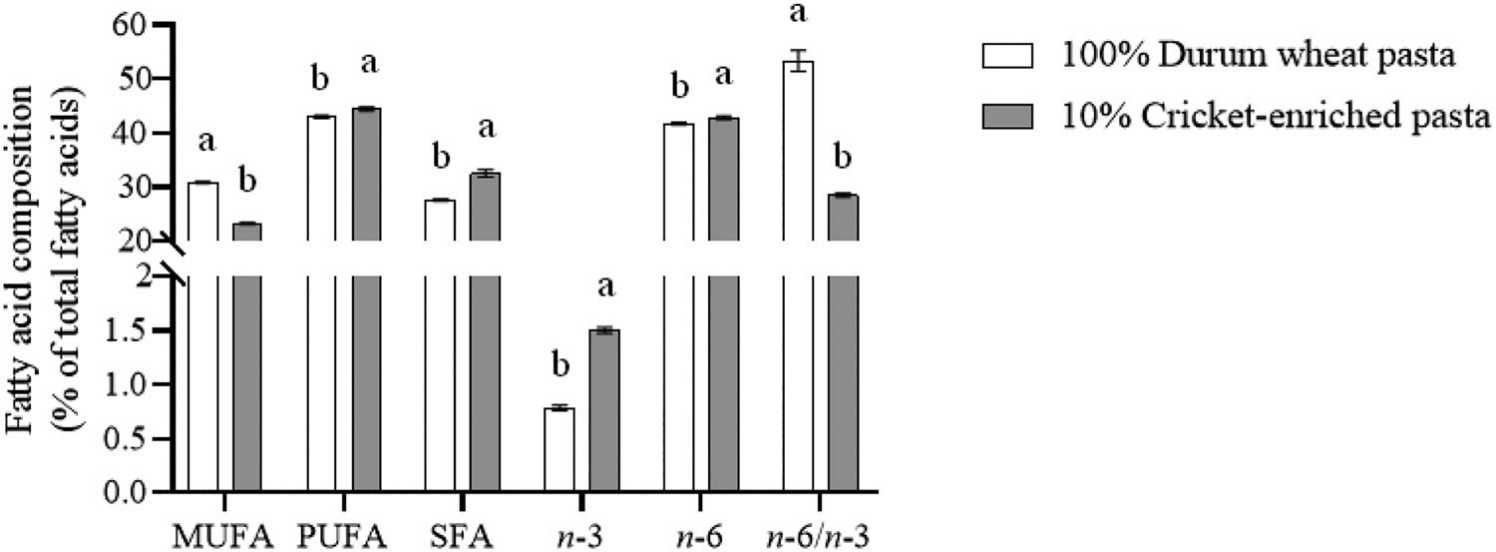
Fatty acid composition (percentage of total fatty acids) of durum wheat and cricket‐enriched pasta. Different letters indicate significant differences (*p* < 0.05) between pasta types. Data are expressed as mean ± standard deviation (*n* = 3 analytical replicates). MUFA, monounsaturated fatty acids; *n*‐3, omega‐3 fatty acids; *n*‐6, omega‐6 fatty acids; PUFA, polyunsaturated fatty acids; SFA, saturated fatty acids.

Total lipid content also increased significantly (*p* < 0.05) in cricket‐enriched pasta (2.50 g/100 g) versus durum wheat pasta (1.26 g/100 g), consistent with previous research (Duda et al. 2019; Ho et al. 2022). The FA profile revealed higher proportions of PUFA (44.5% vs. 43.0%) and SFA (32.5% vs. 27.6%) and lower monounsaturated FAs (MUFA) (23.2% vs. 30.9%) in the cricket‐enriched pasta (Figure 2); these results align with the FA profile of wheat flour (Tavoletti et al. 2021). Notably, PUFA omega‐3 content increased by 48% in the cricket‐enriched pasta, whereas PUFA omega‐6 rose modestly (3%), resulting in a 47% lower omega‐6/omega‐3 ratio (Figure 2). Table S2 highlights higher levels of essential FA, specifically C18:2 linoleic acid (4%) and C18:3 *n*‐3 α‐linolenic acid (55%) in the enriched pasta, reflecting the FA profile of cricket powder (EFSA NDA et al. 2024). From a nutritional perspective, the 47% reduction in the omega‐6/omega‐3 ratio (from 15.4 to 8.1) represents a meaningful improvement, as lower ratios are associated with reduced cardiovascular and inflammatory risks (Simopoulos 2008). Furthermore, this reduction in the omega‐6/omega‐3 ratio is nutritionally relevant when compared to recommended dietary targets. Current recommendations suggest ratios between 4:1 and 5:1 for chronic disease prevention, with Western diets typically ranging from 15:1 to 20:1 (Simopoulos 2008). Although the cricket‐enriched pasta ratio (8.1) still exceeds ideal targets, it represents a substantial step toward recommended values compared to the control pasta (15.4), which falls within the typical Western diet range (Simopoulos 2008). Although the absolute changes in total PUFA appear modest, the substantial increases in omega‐3 content and specifically *n*‐3 α‐linolenic acid enhance the functional value of the enriched pasta. Moreover, docosahexaenoic acid (C22:6 *n*‐3) was detected in the cricket‐enriched pasta (0.05%) but was absent in the durum wheat control, representing an additional nutritional benefit, as DHA is a long‐chain omega‐3 FA with well‐established cardiovascular and neuroprotective effects, including anti‐inflammatory properties, improved endothelial function, and reduced risk of coronary heart disease (Mozaffarian and Wu 2011; Swanson et al. 2012).

Furthermore, total dietary fiber content significantly increased (*p* < 0.05) in the enriched pasta (5.48 g/100 g) compared to the control (2.90 g/100 g) (Figure 1). Although previous studies have not reported dietary fiber content in cricket‐enriched pasta, other insect‐enriched pastas (e.g., grasshopper and mealworm) also showed significant increases in dietary fiber content (Çabuk and Yilmaz 2020).

Conversely, total carbohydrates (61.45 vs. 71.01 g/100 g) and starch content (62.26 vs. 70.00 g/100 g) were significantly lower in the cricket‐enriched pasta (*p* < 0.05) (Figure 1). This is consistent with previous findings (Duda et al. 2019; Ho et al. 2022), even though the starch content had not been directly measured.

Cricket powder is a nutrient‐dense ingredient, rich in protein (66.8%), lipids (16.7%), minerals (4.6%), and dietary fiber (7.6%), and low in carbohydrates (1%–2%) (EFSA NDA et al. 2024). Thus, the observed modifications in pasta composition are consistent with its known profile. Dietary fiber serves as a crucial energy source for gut microbiota (David et al. 2014), and higher intake is associated with reduced risk of colon cancer, metabolic syndrome, and cardiovascular diseases (Martínez et al. 2013; Tap et al. 2015; Jovandaric et al. 2024). The primary fiber component in house crickets is chitin (EFSA NDA et al. 2024), but its effects on gut microbiota remain underexplored.

### 3.2 Kinetics of Starch Hydrolysis

**FIGURE 3:**
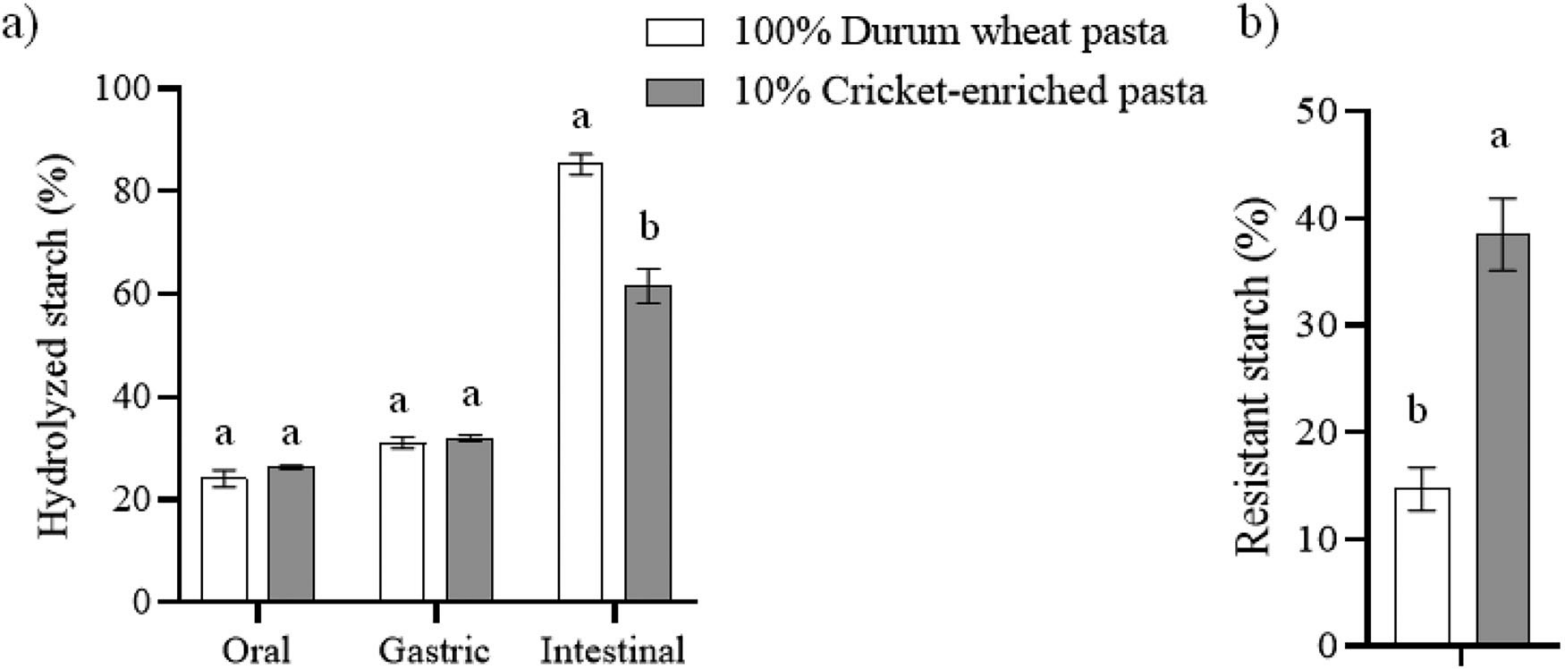
Percentage of (a) hydrolyzed starch after in vitro oral, gastric, and intestinal digestion phases and (b) resistant starch percentage of durum wheat and cricket‐enriched pasta. Different letters indicate significant differences (*p* < 0.05) between pasta types. Data are expressed as mean ± standard deviation (*n* = 3 analytical replicates).

**FIGURE 4:**
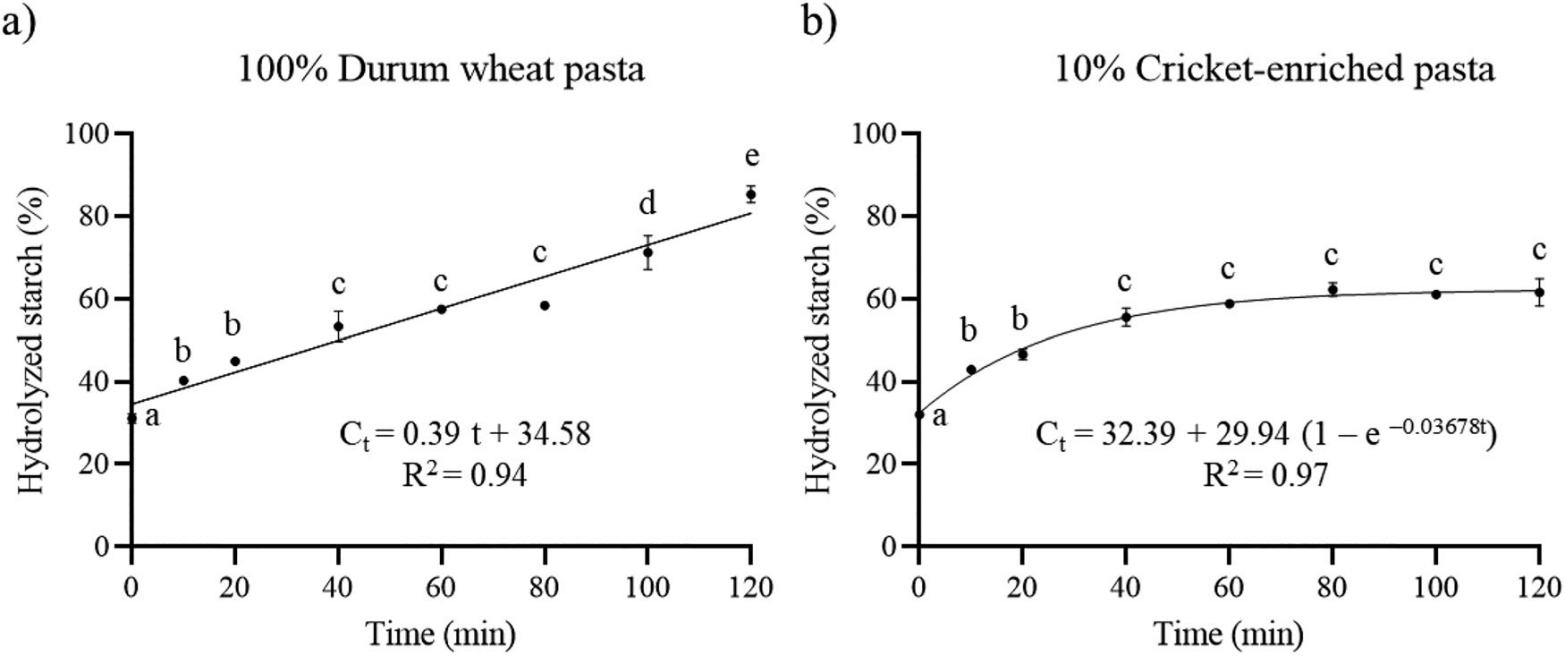
Percentage of hydrolyzed starch during intestinal phase in (a) durum wheat pasta and (b) cricket‐enriched pasta. Data are expressed as mean ± standard deviation (*n* = 3 analytical replicates). Different letters (a–e) indicate statistically significant differences (*p* < 0.05) between the different time points of hydrolyzed starch within the same pasta type.

Given that pasta is a high‐carbohydrate food, the impact of cricket powder enrichment on starch hydrolysis kinetics was assessed, as this can provide valuable insights into potential modifications in the glycemic response within the body. Figure 3 shows the percentage of hydrolyzed starch in relation to the total starch in the sample, after the oral, gastric, and intestinal digestion phases, as well as the percentage of resistant starch. Figure 4 shows the kinetics of starch hydrolysis during the intestinal digestion phase. After the oral phase, no significant differences (*p* > 0.05) were observed in the percentage of hydrolyzed starch between both types of pasta (26.4% vs. 24.1%), as well as after the gastric phase (32.0% vs. 31.1%). At this stage, the hydrolyzed starch reflects the molecular breakdown occurring during both the oral and gastric phases. Previous studies have also reported that approximately 30% of the starch in durum wheat pasta is hydrolyzed by the end of the gastric phase (Freitas and Le Feunteun 2019). As for the intestinal phase, the percentage of hydrolyzed starch in durum wheat pasta increases proportionally over time, following a linear hydrolysis kinetics (*C_t_ = *0.3864 *t + *34.58). However, in the cricket‐enriched pasta, it is observed that from approximately 60 min the percentage of hydrolyzed starch stabilizes, following in this case a first order hydrolysis kinetics (*C_t_ = *32.39 + 29.94 (1 *− *e*^−^* ^0.03678^ *^t^*). This means that cricket‐enriched pasta not only has a lower starch percentage compared to durum wheat pasta, but also that a significantly lower proportion of starch (61.5% vs. 85.3%) is hydrolyzed during in vitro gastrointestinal digestion (*p* < 0.05), thus having a higher proportion of resistant starch (38.5% vs. 14.7%) (*p* < 0.05). Resistant starch promotes the proper functioning of glucose metabolism by exerting a prebiotic effect on the microbiota, leading to the formation of short‐chain FA, mainly butyrate (Li et al. 2024). In addition, the glycemic index of cricket‐enriched pasta is expected to be lower than that of conventional wheat pasta, as other food enriched with 10% cricket‐powder, such as corn tortillas (*A. domesticus*) (Alvarez‐Barajas et al. 2023) and muffins (*Gryllodes sigillatus*) (Zielińska et al. 2021), have shown a 14% reduction in glycemic index compared to their non‐enriched counterparts. This further supports the potential of cricket‐enriched pasta for dietary management in conditions requiring glycemic control, even though in vivo validation is necessary to confirm these effects.

Starch digestibility of *A. domesticus*‐enriched food has been evaluated in 10% enriched corn tortillas, obtaining very similar results to those described in the present work; a lower percentage (16%) of total starch, as well as a higher proportion (27%) of resistant starch, was found in the enriched corn tortillas compared to the non‐enriched ones (Alvarez‐Barajas et al. 2023). Similar results have been observed in other studies on starch digestibility in 10% cricket‐enriched (*G. sigillatus*) muffins (Zielińska et al. 2021). The results could be explained by the higher lipid, protein, and fiber content of the flour‐based foods enriched with cricket powder. Lipids may form amylose–lipid complexes, and cysteine residues from proteins may form disulfide bonds with gelatinized starch after cooking, hindering the action of gastrointestinal enzymes and increasing the proportion of resistant starch (Alvarez‐Barajas et al. 2023). Specifically, proteins can non‐covalently interact with starch through hydrogen bonding, hydrophobic interactions, electrostatic forces, ionic interactions, and van der Waals forces (Chi et al. 2022). These protein–starch interactions restrict starch disorganization, reduce swelling of starch granules, and suppress the accessibility of digestive enzymes to starch granules (Chi et al. 2022; Yang et al. 2023). Moreover, proteins can form continuous networks in the starch matrix that act as physical barriers, blocking the contact between digestive enzymes and starch (Krishnan et al. 2022). Regarding lipids, they interact with starch (particularly amylose) to form starch–lipid complexes that also limit starch swelling and reduce the accessibility of digestive enzymes. The starch–lipid complex promotes the formation of ordered structures, including increased short‐range molecular order, crystallinity, and helical content, which further modulate starch digestibility. The extent of this inhibitory effect depends on lipid characteristics. Lipids with longer chain lengths and higher saturation degrees exhibit stronger resistance to hydrolysis, as they induce stronger hydrophobic interactions and higher thermal stability within the amylose helical cavity (Yang et al. 2023). Additionally, dietary fiber can act as a physical barrier between the enzymes and the food, also causing an increase in resistant starch (Alvarez‐Barajas et al. 2023). In particular, chitin can create a protective layer around starch granules, forming an integrated chitin‐starch network structure that prevents α‐amylase from accessing and binding to starch. The hydrophobic nature of chitin may further limit the availability of water for enzyme‐substrate reactions, thereby reducing overall starch hydrolysis (Bai et al. 2023).

It is important to mention that these interactions do not appear to negatively affect protein digestibility. Although chitin present in the insect exoskeleton has been reported to potentially bind proteins and reduce their digestibility (Rumpold and Schlüter 2013), this effect has not been observed in carbohydrate‐rich food matrices such as cricket‐enriched corn tortillas. In fact, Alvarez‐Barajas et al. (2023) reported a higher percentage of digested protein in cricket‐enriched tortillas compared to control tortillas (91.4% vs. 72.3%), suggesting that the formation of starch–protein complexes selectively impacts starch hydrolysis without compromising protein absorption. However, none of the mentioned studies followed the harmonized INFOGEST 2.0 methodology for gastrointestinal in vitro digestion, which is currently the most recommended standardized protocol for simulating human digestion (Brodkorb et al. 2019). Therefore, our results may offer a more accurate representation of starch digestibility, providing robust insights into the potential impact of cricket‐enriched pasta on glycemic response, which can serve as a foundation for future research endeavors in this promising field.

### 3.3 Total Antioxidant Capacity

**FIGURE 5:**
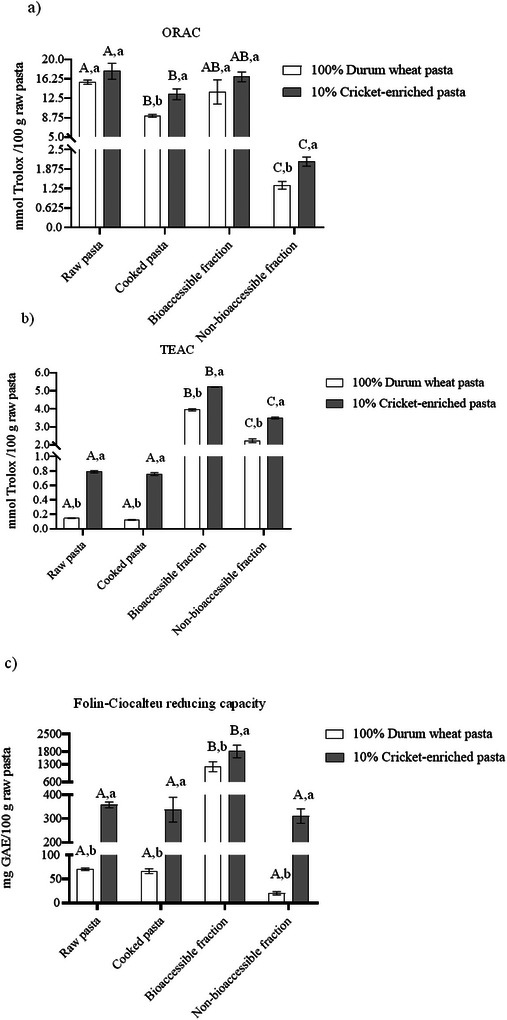
Antioxidant capacity of durum wheat and cricket‐enriched pasta following (a) ORAC, (b) TEAC, and (c) Folin–Ciocalteu reducing capacity methods. Different uppercase letters indicate significant differences (*p* < 0.05) between samples within the same pasta type. Different lowercase letters indicate significant differences (*p* < 0.05) between pasta types. Data are expressed as mean ± standard deviation (*n* = 3 analytical replicates). ORAC, oxygen radical absorbance capacity; TEAC, Trolox equivalent antioxidant capacity.

Antioxidant capacity was evaluated to assess the impact of cricket powder incorporation into durum wheat pasta, as well as to determine the changes induced by cooking and simulated gastrointestinal digestion. As shown in Figure 5, a consistent trend was noted across the three analytical methods used (ORAC, TEAC, and Folin–Ciocalteu reducing capacity). Cricket‐enriched pasta exhibited significantly higher (*p* < 0.05) antioxidant capacity than conventional durum wheat pasta in all sample types analyzed: raw (14%–437%), cooked (45%–513%), BF (21%–50%), and NBF (56%–1437%). An exception was observed in the ORAC assay, where no significant differences (*p* > 0.05) were detected between the two pasta types in the BF (16.62 vs. 13.72 mmol Trolox/100 g) or the raw pasta (17.72 vs. 15.60 mmol Trolox/100 g), even though a trend toward higher antioxidant capacity was observed in the cricket‐enriched pastas. These findings align with previous studies that reported increased antioxidant capacity in 10% cricket powder‐enriched foods, such as rice crackers (TEAC: 250% increase; *A. domesticus*) (Suga et al. 2023), muffins (TEAC: 118% increase; *G. sigillatus*) (Zielińska et al. 2021), and millet pasta (Folin–Ciocalteu: 102%; *G. sigillatus*) (Jakab et al. 2020). Cricket powder is recognized for its notably high antioxidant potential (up to five times that of fresh orange juice: 0.40 vs. 2.37 mmol Trolox/100 g) and often surpasses antioxidant capacity of other edible insects (mmol Trolox/100 g) such as mealworms (0.89), African caterpillars (1.43), mini crickets (0.85), buffalo worms (0.82), scolopendra (0.78), black ants (0.57), and palm worm (0.55) (Di Mattia et al. 2019). Notably, even at relatively low enrichment levels (≤10%), cricket powder can significantly enhance the antioxidant profile of fortified foods, as evidenced in our study.

Regarding the effects of cooking, antioxidant capacity remained largely unaffected in both pasta types across all methods, except for the ORAC assay. In this case, cooking resulted in a significant reduction in antioxidant capacity, with a 41% decrease in durum wheat pasta compared to a smaller 25% reduction in cricket‐enriched pasta (Figure 5a). This attenuation may be due to the solubilization of antioxidant compounds in cooking water. However, the higher fiber content in the cricket‐enriched pasta may help mitigating these losses, as dietary fiber can form a gel‐like matrix that entraps antioxidants and reduces their leaching, according to previous studies (Palafox‐Carlos et al. 2011).

Simulated gastrointestinal digestion further influenced antioxidant capacity. The BF exhibited a 17‐ to 32‐fold increase in antioxidant capacity for durum wheat pasta and a 5‐ to 7‐fold increase for cricket‐enriched pasta when compared to raw and cooked samples, respectively, with the exception of the ORAC assay. In fact, in the latter, no significant differences were noted between the BF and raw pasta; however, the cooked pasta and NBF exhibited diminished antioxidant capacity, with reductions of 42% and 91% for cooked and NBF durum wheat pasta and 25% and 88% for cooked and NBF cricket‐enriched pasta, respectively, compared to raw pasta (*p* < 0.05). The observed differences among assays may stem from their distinct reaction mechanisms: ORAC is based on hydrogen atom transfer, whereas both the TEAC and Folin–Ciocalteu methods rely on electron transfer reactions (Prior et al. 2005). This divergence could account for the discrepancies in antioxidant capacity measurements across sample types. Additionally, gastrointestinal digestion likely hydrolyzes proteins, releasing bioactive peptides with antioxidant properties. Previous studies have identified such peptides in digested cricket protein (Zielińska et al. 2018). As proteins and peptides are known reducing agents (Esfandi et al. 2019), it is consistent that the BF would show elevated antioxidant capacity in methods that measure electron transfer, but not necessarily in ORAC.

From a physiological perspective, the distinction between the BF and NBF has important health implications. The BF represents compounds that are solubilized during digestion and potentially available for intestinal absorption. The substantial increases observed in the BF suggest that gastrointestinal digestion enhances the release and availability of antioxidant compounds that could exert systemic protective effects against oxidative stress after entering the bloodstream (Palafox‐Carlos et al. 2011). Conversely, the NBF, which also exhibited antioxidant capacity increases in cricket‐enriched pasta, represents unabsorbed compounds that reach the colon. These compounds may exert local antioxidant effects in the colon, potentially contributing to gut health by reducing oxidative damage to colonocytes and modulating inflammatory processes in the intestinal mucosa (Rajoka et al. 2021). This dual functionality suggests that cricket‐enriched pasta may provide comprehensive antioxidant benefits throughout the gastrointestinal tract.

It should be noted that the elevated antioxidant capacity observed in cricket‐enriched pasta may arise from multiple sources: endogenous antioxidants naturally present in cricket powder (such as free amino acids, peptides, and phenolic compounds), as well as neo‐formed antioxidant compounds generated through Maillard reactions during cooking and protein hydrolysis during gastrointestinal digestion. Future research should identify the primary contributors to antioxidant activity in cricket‐enriched pasta, both in the raw and cooked product, as well as in the BF, to better understand the mechanisms through which cricket‐enriched pasta exert antioxidant capacity and could confer potential health benefits related to oxidative stress regulation.

### 3.4 Technological Properties

**FIGURE 6:**
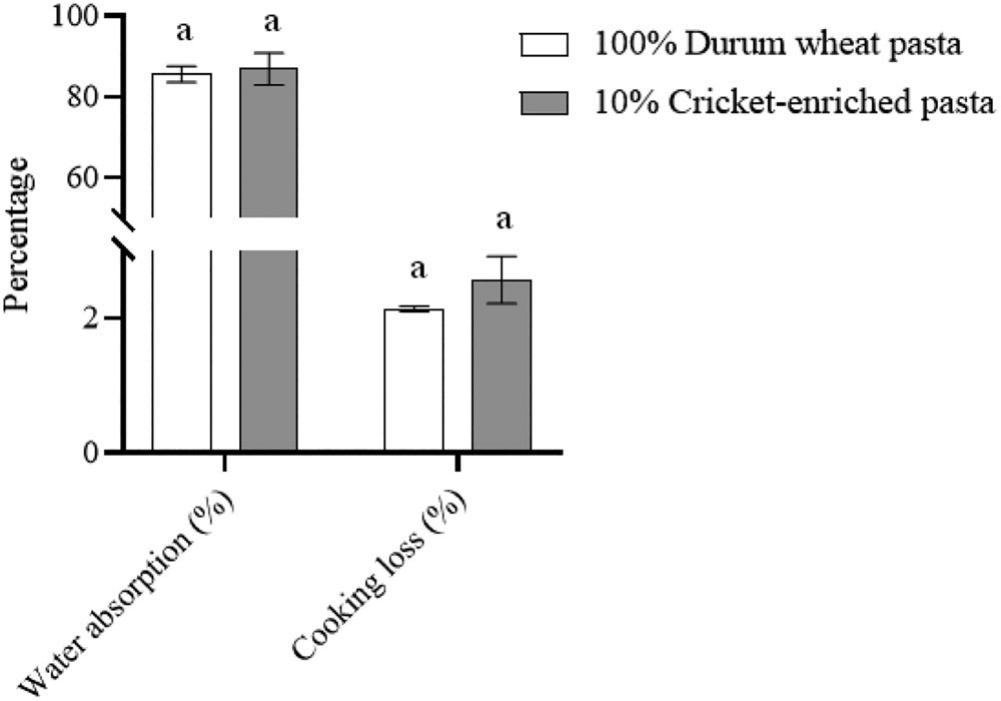
Water absorption and cooking loss of durum wheat and cricket‐enriched pasta. Different letters indicate significant differences (*p* < 0.05) between pasta types. Data are expressed as mean ± standard deviation (*n* = 3 analytical replicates).

Culinary properties were determined to assess whether cricket enrichment of durum wheat pasta could negatively affect them. The culinary properties evaluated were WA (85.61% vs. 86.94%) and cooking loss (2.14% vs. 2.57%) for durum wheat and cricket‐enriched pasta, respectively (Figure 6). In both cases, no significant differences were observed between the two types of pasta (*p *> 0.05). Ho et al. (2022) also observed no differences in WA and cooking loss after the addition of the cricket powder (*A. domesticus*) to wheat pasta. However, other studies have reported increased WA and cooking loss in cricket‐enriched (*A. domesticus*) pasta compared to non‐enriched wheat pasta (Pasini et al. 2022; Bresciani et al. 2022), whereas Duda et al. (2019) observed significant decreases. These discrepancies could be attributed to differences in enrichment levels (5%–10% and 14%, respectively) or the type of cricket ingredient used (cricket powder and protein extract, respectively).

**FIGURE 7:**
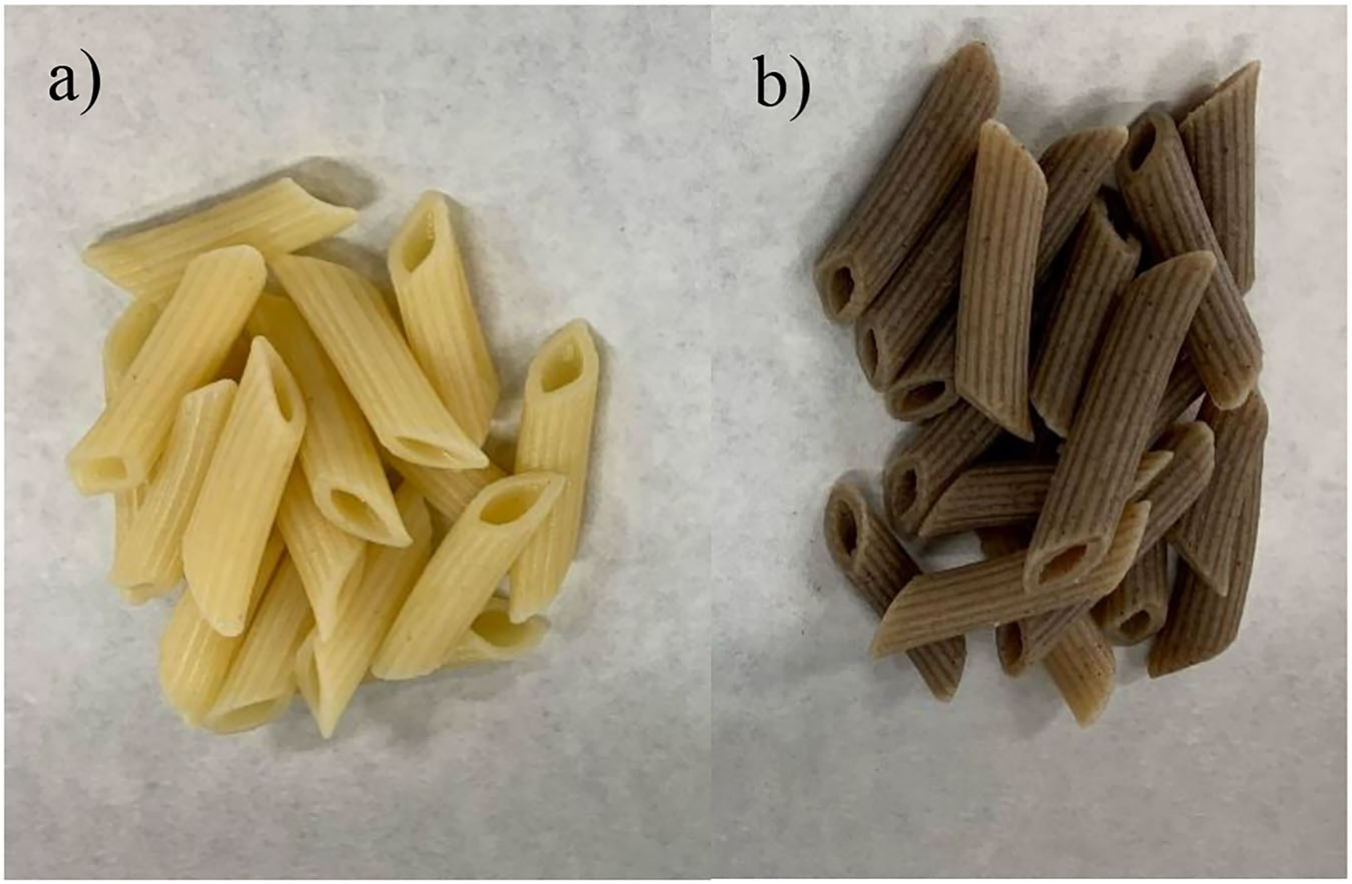
(a) 100% durum wheat and (b) 10% cricket‐enriched pasta.

**FIGURE 8:**
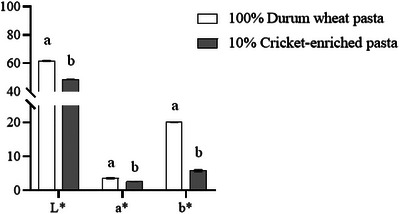
Colorimetric properties of durum wheat and cricket‐enriched pasta. Different letters indicate significant differences (*p* < 0.05) between pasta. Data are expressed as mean ± standard deviation (*n* = 3 analytical replicates).

Color is a critical attribute influencing consumer choices in the pasta market (Sajdakowska et al. 2021). The cricket‐enriched pasta displayed a distinctly darker color relative to the durum wheat pasta (Figure 7). Darker shades are often associated with high‐fiber pasta, which can affect overall acceptance. In general, consumers tend to show lower acceptance for modified pasta with a more intense, darker color, as it is often perceived to have a bitter or more acidic taste. However, acceptance increases among consumers who regularly purchase nonconventional pasta varieties (Sajdakowska et al. 2021). Additionally, colorimetric properties were determined using the CIELAB scale on durum wheat and cricket‐enriched (Figure 8). The L* value (ranging from black to white) was significantly different (*p* < 0.05) between the two pasta types (48.75 vs. 61.80 cricket‐enriched and durum wheat pasta, respectively). The a* value (ranging from red to green) also showed significant differences (*p* < 0.05) among both samples, with cricket‐enriched pasta presenting the lowest value (2.67) compared to durum wheat pasta (3.63). Finally, for the b* value (ranging from blue to yellow), significant differences (*p* < 0.05) were also observed; cricket‐enriched pasta (5.87) and durum wheat pasta (20.11). Overall, the colorimetric determinations highlight the notable differences among both pasta types, as reflected in the Δ*E*_Lab_ calculation (19.34). Δ*E*_Lab_ values of 5 or above imply that two different colors are perceptible by an untrained observer (Biró et al. 2019). According to the results, the colorimetric changes after the addition of cricket powder to wheat pasta are characterized by a decrease in lightness and an increase in red and blue colors. This same trend has been reported in other studies on cricket‐enriched pasta (Duda et al. 2019; Jakab et al. 2020).

**FIGURE 9:**
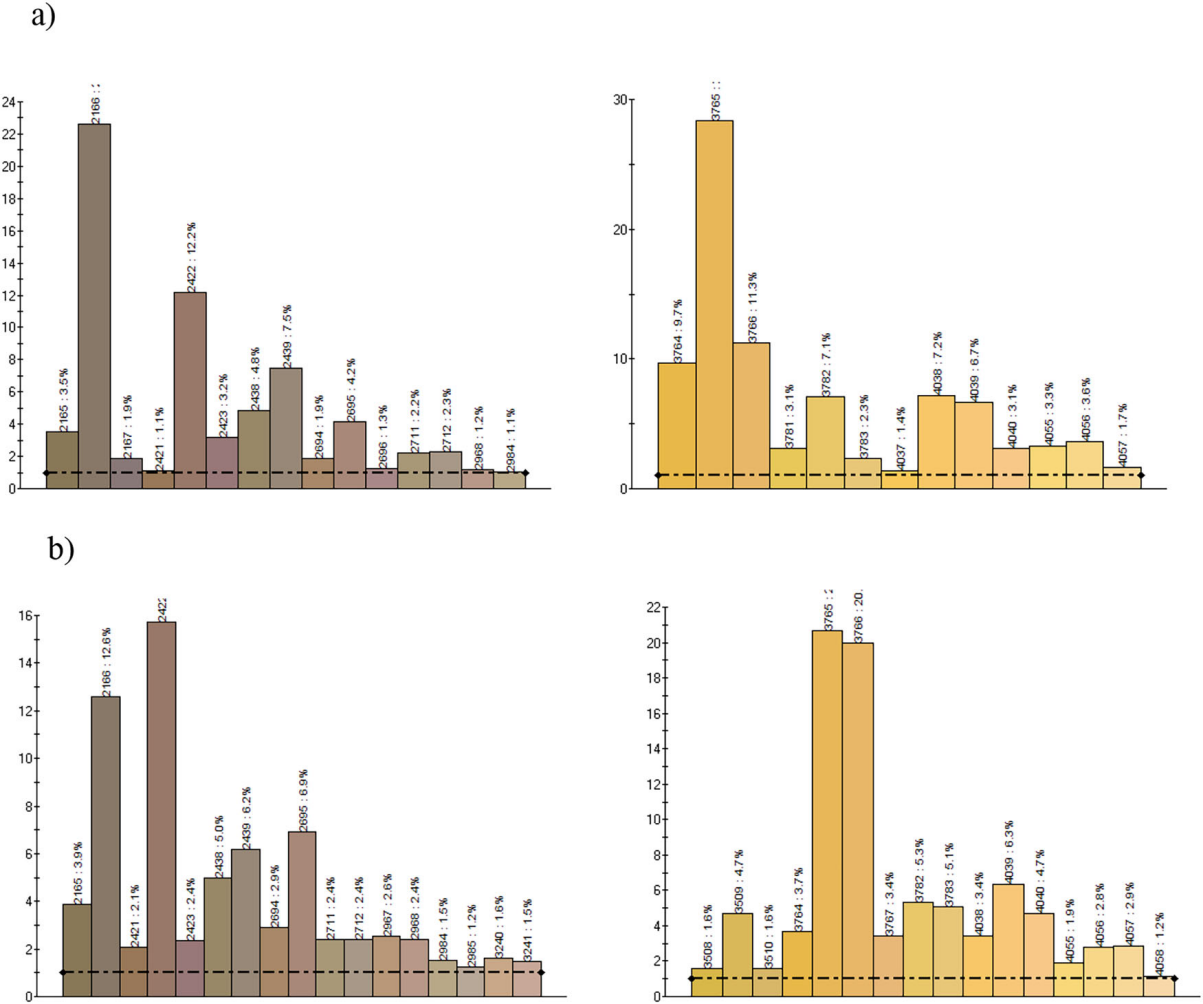
Spectra of cricket‐enriched pasta (left) and durum wheat pasta (right) obtained using an electronic eye. (a) Spectrum of raw pasta; (b) spectrum of cooked pasta. Data are expressed as mean ± standard deviation (*n* = 3 analytical replicates).

The visual appearance of pasta samples was also evaluated using an electronic eye system, which enables rapid and high‐resolution image acquisition and analysis of color‐related parameters through artificial vision technology. The data indicated that each formulation possessed a distinct chromatic profile. In particular, pasta enriched with 10% cricket powder was characterized by color variables associated with darker, brownish hues (Figure 9). This confirms the strong visual impact of insect powder on the appearance of the final product, especially after cooking. Durum wheat pasta samples displayed a color profile ranging from yellow to ochre, reflecting the typical visual characteristics of traditional 100% wheat pasta (Figure 9). Overall, the results demonstrated that the addition of cricket powder significantly influenced pasta color, allowing the electronic eye system to effectively discriminate among the different samples.

The consistency between colorimetric and image analysis confirms the distinctive chromatic profile of cricket‐enriched pasta. These visual characteristics have important implications for consumer acceptance, as the darker appearance may function as a recognizable indicator of enhanced nutritional value, potentially appealing to health‐conscious consumers and regular purchasers of functional pasta, while possibly requiring tailored marketing approaches to address perceptions among consumers less familiar with fortified pasta products (Sajdakowska et al. 2021).

### 3.5 Limitations

Although this study provides novel insights into the starch hydrolysis kinetics and antioxidant capacity of cricket‐enriched pasta following simulated gastrointestinal digestion, several limitations should be acknowledged. Although observed differences between samples are primarily ascribable to cricket powder incorporation, potential variations in manufacturing processes (e.g., formulation, extrusion, and drying) cannot be entirely ruled out as two pasta types were obtained from different suppliers.

Furthermore, a single enrichment level (10%) was evaluated, selected based on literature demonstrating an optimal nutritional‐sensory balance (Duda et al. 2019). Although linear dose–response relationships have been documented for nutritional improvements and functional properties in cricket‐enriched products (Duda et al. 2019; Zielińska et al. 2021; Álvarez‐Barajas et al. 2023), investigating multiple levels would have provided valuable additional information, particularly regarding the impact on starch hydrolysis kinetics. Future research should address dose–response effects to establish optimal formulation guidelines.

Another limitation concerns the absence of sensory evaluation. Although previous research notes acceptable consumer acceptance of 10% cricket‐enriched durum wheat pasta (Duda et al. 2019), a comprehensive sensory analysis would have provided direct evidence of the implications of the observed color and compositional changes. Future investigations should integrate sensory, nutritional, and functional assessments for a holistic product evaluation. Additionally, amino acid composition and protein digestibility were not directly assessed. Although literature supports high insect protein digestibility (76%–96%) and unhindered absorption despite starch–protein complexes formation (Álvarez‐Barajas et al. 2023), direct measurement of bioaccessibility, amino acids, and digestibility would provide more comprehensive nutritional profiling.

Finally, observed starch kinetics suggest glycemic benefits, but these rely on in vitro data. As in vitro models do not fully replicate human physiology, health benefits regarding glycemic control require validation through preclinical and clinical studies. Despite these limitations, this study represents the first application of the standardized INFOGEST 2.0 protocol to cricket‐enriched pasta, providing novel evidence of altered starch digestion kinetics and enhanced antioxidant capacity following simulated gastrointestinal digestion. These findings establish a foundation for future comprehensive investigations into the health implications of insect‐fortified foods.

## 4 Conclusions

This study represents the first investigation into both starch hydrolysis kinetics and antioxidant capacity following simulated gastrointestinal digestion in cricket‐enriched pasta. Incorporation of 10% cricket powder preserved the technological properties of pasta while significantly enhancing its nutritional profile, including increased protein, mineral content, dietary fiber, and an improved FA composition. The observed first‐order starch hydrolysis kinetics, along with the significantly higher resistant starch content, suggests potential benefits for glycemic response modulation. Moreover, cricket‐enriched pasta exhibited significantly greater antioxidant capacity, indicating the presence of enhanced antioxidant compounds released during digestion and potential bioactivity after intestinal absorption. The altered color profile, which more closely resembles that of whole grain pasta containing between 5% and 30% of bran, may also increase consumer acceptance, particularly among environmentally and health‐conscious individuals. Overall, these findings support cricket‐enriched pasta as a promising candidate for functional food development, demonstrating significant nutritional improvements and suggesting potential health benefits that require in vivo validation. Future research should initially focus on acceptability of cricket pasta at the sensory level and preclinical studies using in vitro and in vivo models to elucidate the underlying mechanisms of action, followed by well‐designed human clinical trials to confirm these effects under physiological conditions. Such evidence could foster the integration of insect‐based functional foods into mainstream dietary patterns and support the transition toward a more sustainable food system capable of meeting the nutritional needs of a growing global population.

## Author Contributions

**Leonardo Musto**: investigation, writing – original draft, formal analysis. **Mussa Makran**: investigation, writing – original draft, formal analysis. **Dario Mercatante**: investigation, writing – original draft, formal analysis. **Ivan Albano**: writing – review and editing. **Maria Teresa Rodriguez‐Estrada**: conceptualization, funding acquisition, writing – review and editing, project administration, supervision. **Antonio Cilla**: conceptualization, funding acquisition, writing – review and editing, project administration, supervision. **Guadalupe Garcia‐llatas**: conceptualization, funding acquisition, writing – review and editing, project administration, supervision.

## Conflicts of Interest

The authors declare no conflicts of interest.

## Supporting information




**Supplementary Materials**: jfds70861‐sup‐0001‐SuppMat.docx

## Data Availability

The data presented in this study are available on request from the corresponding author.
