# Supplementary material for: Fortified Pasta With Cricket (Acheta domesticus) Powder: Impact of an Alternative Protein Source on Pasta Nutritional, Functional, and Technological Properties
Source: J Food Sci. 2026 Jan 18;91(1):e70861. doi: 10.1111/1750-3841.70861 (PMC12813587; doi:10.1111/1750-3841.70861)
Supplement: Supplementary file 1 — Supplementary Materials: jfds70861‐sup‐0001‐SuppMat.docx [file JFDS-91-0-s001.docx]

**2.3S. Proximate composition and starch content**

The proximate composition of 100% durum wheat pasta and cricket powder-enriched pasta was analyzed. Prior to analysis, both pasta samples were ground into a fine powder using an electric coffee grinder (VeoHome, Les Étilleux, France). All analyses were performed in triplicate (*n* = 3) and results were expressed on a wet weight basis.

Moisture content was determined according to American Association of Cereal Chemists (AACC) Methods 44-19.01 by drying approximately 3 g of sample in an air oven (ULE 500, Memmert, Büchenbach, Germany) at 135 °C to constant weight.

Ash content was measured following AACC Method 08-12.01. Approximately 3 g of sample was incinerated in a muffle furnace (Heraeus incineration oven type K1253-KAT, Heraeus Holding GmbH, Hanau, Germany) at 600 °C for 8 h.

Crude protein was determined according to the Kjeldahl method (AACC Method 46-12.01). Approximately 250 mg of ground sample was digested with concentrated sulfuric acid (95%) in the presence of a catalyst mixture (potassium sulfate and copper sulfate) until a clear solution was obtained. The digest was then neutralized and distilled (using a Kjeltec KT 200, Labtec Line, FOSS, Warszawa, Poland) with sodium hydroxide (50%, w/v) and the released ammonia was collected in a boric acid solution (4%, p/v). The distillate was subsequently titrated with standardized hydrochloric acid (HCl, 0.1 N) to determine total nitrogen content. Protein content was calculated by multiplying the nitrogen value by a conversion factor of 6.25.

Total fat content was determined following a modified Soxhlet extraction method based on AOAC Official Method 14.059. Approximately 10 g of sample were hydrolyzed with 100 mL of 3 N HCl in a water bath at 100 °C for 1 h to release bound lipids. After cooling, the mixture was filtered through a Whatman No. 2 filter paper previously moistened with distilled water, and the residue was washed repeatedly with distilled water until the filtrate reached neutral pH. The solid residue was then oven-dried at 100 °C to constant weight and transferred to a Soxhlet extraction thimble. Lipids were extracted with diethyl ether for 5 h using a conventional Soxhlet apparatus (bath temperature 80 °C). The solvent was removed by evaporation, and the lipid fraction was dried at 50 °C for 1 h, cooled in a desiccator, and weighed.

Total dietary fiber was determined by an enzymatic–gravimetric method using a commercial assay kit (TDF100A, Supelco), following AACC Method 32-05.01. Approximately 3 g of sample were enzymatically digested sequentially with α-amylase, protease, and amyloglucosidase to remove starch and protein components. The soluble and insoluble fiber fractions were then precipitated with 95% ethanol, filtered through crucibles containing Celite, and washed successively with 78% ethanol and 95% acetone. The residues were dried at 105 °C to constant weight. Protein and ash contents of duplicate samples were determined (by the Kjeldahl method and incineration at 525 °C for 5 h, respectively) and subtracted from the total residue to obtain the total dietary fiber content.

Total starch content was determined according to AOAC Official Method 996.11 using a Megazyme enzymatic kit (K-TSTA 04/2009). Approximately 100 mg of sample were gelatinized with 2 M KOH, neutralized with sodium acetate buffer (pH 3.8), and hydrolyzed with α-amylase and amyloglucosidase at 50 °C. The released glucose was quantified colorimetrically using GOPOD reagent, and absorbance was read at 510 nm.

Carbohydrate content was estimated by difference, subtracting the measured values of moisture, fat, protein, ash, and dietary fiber from 100%.

All results were expressed as g/100 g of raw sample on a wet basis.

**Table S1**. Proximate composition of 100% durum wheat pasta and 10% cricket-enriched pasta.

|  | **Proximate composition (g/100 g raw pasta)** | |
| --- | --- | --- |
|  | **100% durum wheat** | **10% cricket-enriched** |
| **Moisture** | 10.08 ± 0.05^a^ | 10.10 ± 0.06^a^ |
| **Protein** | 13.88 ± 0.28^a^ | 18.87 ± 0.32^b^ |
| **Ash** | 0.88 ± 0.06^a^ | 1.02 ± 0.02^b^ |
| **Lipid** | 1.26 ± 0.07^a^ | 2.50 ± 0.16^b^ |
| **Carbohydrate** | 71.01 ± 0.80^a^ | 62.01 ± 0.71^b^ |
| **Total dietary fiber** | 2.90 ± 0.29^a^ | 5.48 ± 0.13^b^ |

Data are shown as mean ± standard deviation (*n* = 3). Different letters (a-b) indicate significant differences (*p* < 0.05) between pasta types.

**Table S2**. Fatty acid composition (% of total fatty acids) of 100% durum wheat and 10% cricket-enriched pasta.

| **Fatty acid (% of total fatty acids)** | | **Sample** | |
| --- | --- | --- | --- |
|  |  | **Durum wheat pasta** | **Cricket-enriched pasta** |
| **Lauric acid** | **C12:0** | 0.11 ± 0.01^b^ | 0.16 ± 0.00^a^ |
| **Myristic acid** | **C14:0** | 0.73 ± 0.01^a^ | 0.74 ± 0.05^a^ |
| **Pentadecanoic acid** | **C15:0** | 0.10 ± 0.02^a^ | 0.07±0.01^a^ |
| **Palmitic acid** | **C16:0** | 21.09 ± 0.10^b^ | 23.77 ± 0.81^a^ |
| **Palmitoleic acid (*n*-9)** | **C16:1 *n*-9** | 0.18 ± 0.03^a^ | 0.30 ± 0.01^b^ |
| **Palmitoleic acid (*n*-7)** | **C16:1 *n*-7** | 0.53 ± 0.02^a^ | 0.45 ± 0.03^b^ |
| **Heptadecanoic acid** | **C17:0** | 0.12 ± 0.01^b^ | 0.17 ± 0.01^a^ |
| **Stearic acid** | **C18:0** | 1.98 ± 0.03^b^ | 7.30 ± 0.35^a^ |
| **Oleic acid** | **C18:1 c*is-*9** | 30.07 ± 0.14^a^ | 22.38 ± 0.20^b^ |
| **Linoleic acid** | **C18:2 *cis*-9,12** | 41.44 ± 0.29^b^ | 42.44 ± 0.34^a^ |
| **α-linolenic acid** | **C18:3 α** | 0.59 ± 0.02^b^ | 1.34 ± 0.04^a^ |
| **Arachidic acid** | **C20:0** | 3.13 ± 0.09^a^ | 0.23 ± 0.03^b^ |
| **Eicosenoic acid** | **C20:1 *n*-11** | 0.09 ± 0.00^b^ | 0.12 ± 0.01^a^ |
| **Eicosadienoic acid** | **C20:2 *n*-6** | 0.10 ± 0.00^a^ | 0.11 ± 0.01^a^ |
| **Docosanoic acid** | **C22:0** | 0.36 ± 0.02^a^ | 0.09 ± 0.01^b^ |
| **Arachidonic acid** | **C20:4 *n*-6** | 0.22 ± 0.02^b^ | 0.30 ± 0.02^a^ |
| **Eicosapentaenoic acid** | **C20:5 *n*-3** | 0.47 ± 0.01^a^ | 0.12 ± 0.01^b^ |
| **Docosapentaenoic acid** | **C22:5 *n*-3** | 0.19 ± 0.02^a^ | 0.11 ± 0.01^b^ |
| **Docosahexaenoic acid** | **C22:6 *n*-3** | n.d.^b^ | 0.05 ± 0.00^a^ |

Different letters indicate significant differences (*p* < 0.05) between pasta types for each fatty acid. N.d., not detected.
